# Fabrication of Adipose-Derived Stem Cell-Based Self-Assembled Scaffold under Hypoxia and Mechanical Stimulation for Urethral Tissue Engineering

**DOI:** 10.3390/ijms22073350

**Published:** 2021-03-25

**Authors:** Zahra Rashidbenam, Mohd Hafidzul Jasman, Guan Hee Tan, Eng Hong Goh, Xeng Inn Fam, Christopher Chee Kong Ho, Zulkifli Md Zainuddin, Reynu Rajan, Rizal Abdul Rani, Fatimah Mohd Nor, Mohamad Aznan Shuhaili, Nik Ritza Kosai, Farrah Hani Imran, Min Hwei Ng

**Affiliations:** 1 Centre for Tissue Engineering and Regenerative Medicine, Universiti Kebangsaan Malaysia Medical Centre, 12th Floor, Clinical Block, Jalan Yaacob Latif, Bandar Tun Razak, Cheras, Kuala Lumpur 56000, Malaysia; zahra.rashidbenam@gmail.com; 2 Clinical Skills Learning and Simulation Unit, Department of Medical Education, Universiti Kebangsaan Malaysia Medical Centre, Jalan Yaacob Latif, Bandar Tun Razak, Cheras, Kuala Lumpur 56000, Malaysia; mohdhafidzuljasman@ukm.edu.my; 3 Urology Unit, Department of Surgery, Universiti Kebangsaan Malaysia Medical Centre, 8th Floor, Clinical Block, Jalan Yaacob Latif, Bandar Tun Razak, Cheras, Kuala Lumpur 56000, Malaysia; mrtan.guanhee@gmail.com (G.H.T.); bobby.goh@hotmail.com (E.H.G.); xenginn@gmail.com (X.I.F.); zuluro@ppukm.ukm.edu.my (Z.M.Z.); 4 School of Medicine, Taylor’s University, No. 1 Jalan Taylor’s, Subang Jaya 47500, Selangor Darul Ehsan, Malaysia; chrisckho2002@yahoo.com; 5 Minimally Invasive Upper Gastrointestinal and Bariatric Surgery Unit, Department of Surgery, Universiti Kebangsaan Malaysia Medical Centre, 8th Floor, Clinical Block, Jalan Yaacob Latif, Bandar Tun Razak, Cheras, Kuala Lumpur 56000, Malaysia; dr.reynu@gmail.com (R.R.); mrdocnan@gmail.com (M.A.S.); nikkosai@yahoo.co.uk (N.R.K.); 6 Arthoplasty Unit, Department of Orthopaedics and Traumatology Surgery, Universiti Kebangsaan Malaysia Medical Centre, 9th Floor, Clinical Block, Jalan Yaacob Latif, Bandar Tun Razak, Cheras, Kuala Lumpur 56000, Malaysia; rizal@ppukm.ukm.edu.my; 7 Plastic and Reconstructive Surgery Unit, Department of Surgery, Universiti Kebangsaan Malaysia Medical Centre, Clinical Block, 8th Floor, Jalan Yaacob Latif, Bandar Tun Razak, Cheras, Kuala Lumpur 56000, Malaysia; drfatimahnor@gmail.com (F.M.N.); farrahhani@gmail.com (F.H.I.)

**Keywords:** adipose-derived stem cell, self-assembled scaffold, extracellular matrix, hypoxic condition, tissue engineering, autologous urethral graft, allogeneic urethral graft, urethral reconstruction

## Abstract

Long urethral strictures are often treated with autologous genital skin and buccal mucosa grafts; however, risk of hair ingrowth and donor site morbidity, restrict their application. To overcome this, we introduced a tissue-engineered human urethra comprising adipose-derived stem cell (ASC)-based self-assembled scaffold, human urothelial cells (UCs) and smooth muscle cells (SMCs). ASCs were cultured with ascorbic acid to stimulate extracellular matrix (ECM) production. The scaffold (ECM) was stained with collagen type-I antibody and the thickness was measured under a confocal microscope. Results showed that the thickest scaffold (28.06 ± 0.59 μm) was achieved with 3 × 10^4^ cells/cm^2^ seeding density, 100 μg/mL ascorbic acid concentration under hypoxic and dynamic culture condition. The biocompatibility assessment showed that UCs and SMCs seeded on the scaffold could proliferate and maintain the expression of their markers (CK7, CK20, UPIa, and UPII) and (α-SMA, MHC and Smootheline), respectively, after 14 days of in vitro culture. ECM gene expression analysis showed that the ASC and dermal fibroblast-based scaffolds (control) were comparable. The ASC-based scaffold can be handled and removed from the plate. This suggests that multiple layers of scaffold can be stacked to form the urothelium (seeded with UCs), submucosal layer (ASCs only), and smooth muscle layer (seeded with SMCs) and has the potential to be developed into a fully functional human urethra for urethral reconstructive surgeries.

## 1. Introduction

Currently, the gold standard grafts used by urethral reconstructive surgeons to treat urethral stricture are autologous grafts from buccal mucosa or genital skin. Even though the buccal mucosa has suitable elasticity and in that sense is suitable for repairing a wide range of urethral strictures in terms of length [1], the complications arising from the application of such grafts like nerve damage and bleeding, and the fact that the composition and anatomy of the grafts differ from that of urethral tissue, are areas of concern. Although, skin grafts are readily used in genitourinary reconstructive surgery, their application can nevertheless lead to complications such as infection due to hair growth, and stone formation in the neourethra. Moreover, there are limitations in utilizing the non-hairy skin of patients with lichen sclerosis, as it can lead to graft failure in the long-term, due to fibrosis [2,3,4,5,6].

Tissue engineering techniques have recently emerged as an alternative approach for treating short and long urethral defects via the construction of an implant or graft that can be used in urethral replacement or reconstruction. So far different types of cells, including progenitor cells, i.e., autologous urothelial cells (UCs) [7], mesenchymal stem cells [8], and induced pluripotent stem cells [9], and biomaterials such as acellular tissue matrices (i.e., bladder submucosa matrix) [10], and synthetic polymers (i.e., poly lactic-co-glycolic acid) [11], have been investigated for their applicability in tissue engineering of urethral graft. However there still exist unresolved issues such as risk of immunogenic reactions [12], ethical concerns [13], poor quality of cells from unhealthy tissue or donor and complications of clinical translation. Therefore, the lack of a desirable engineered tissue constructs to be used as an implant for treating urethral stricture in any safe and practicable manner yet remains as a challenge in this field.

Among strategies in tissue engineering of urethra reviewed elsewhere [6], constructing of urethral tissue using self-assembly approach happens to be promising. Self-assembled scaffold production as a replacement to human connective tissue was first introduced by Dr. Francois A. Auger’s team and it is based on production and deposition of extracellular matrix (ECM) by dermal fibroblasts (DFs) [14] or adipose-derived stem cells (ASCs) [15] under the influence of ascorbic acid. Therefore, DF or ASC-based self-assembled scaffolds are considered as cell-laden scaffolds.

Application of DF-based self-assembled scaffold for tissue engineering of genitourinary tubular graft was first introduced by Magnan et al. (2009) and it was shown that the scaffold is capable of supporting the pluristratified urothelium formation and expression of keratin (CK) 8/18, a specific marker of transitional epithelium by the UCs seeded on the scaffold [16]. In next attempts, it was also proven that DF-based self-assembled scaffold, when it is placed under dynamic condition is able to support the expression of uroplakin (UP) II and CK20 by seeded UCs [17] and develop feature of impermeability to urea [18].

In an interesting study, ability of the self-assembled scaffold in supporting the phenotype of terminally differentiated UCs and the composition of ECM produced by DFs, ASCs and hybrid DF-ASC-based self-assembled scaffolds were compared with one another. All three types of self-assembled scaffolds exhibited almost identical expression of collagen type I, III, fibronectin, and laminin in the ECM. However, expression of markers UPIb, II and III and tight junction marker ZO-1, representing development of terminally differentiated and functional urothelium were detected only in UCs which were seeded on DF-based and hybrid DF-ASC-based self-assembled scaffolds. The absence of laminin 5 expression in ASC-based self-assembled scaffold as opposed to DF-based and hybrid scaffolds was believed to be the contributing factor in inability of ASC-based self-assembled scaffold in supporting the phenotype of terminally differentiate UCs [19].

Previous studies showed that DF and ASC-based self-assembled scaffolds both, exhibit suitable mechanical properties including burst pressure [16], circumferential strength and suture retention strength [15], to be used as a graft in reconstructive surgeries. Even though ASC-based self-assembled scaffold presented significantly better compliance as compared to DF-based scaffold, both DF and ASC were found to develop self-assembled scaffolds with comparable thicknesses [15] and were sturdy enough to be manipulated by a surgeon [15,16].

Although DF-based self-assembled scaffold had been proven to be suitable to be used for tissue engineering of human urethra, it may promote scarring due to fibrosis; and in that sense therefore, ASC-based self-assembled scaffold has privilege over the DF-based one. Moreover, using ASCs for production of self-assembled scaffold to be used for tissue engineering of human urethra offers several advantages over DFs; first, all components of human urethra (UCs, SMCs and connective tissue) can potentially be produced from a single source of tissue (i.e., adipose tissue) as there exist established methods for in vitro differentiation of ASCs into SMCs [20] and UCs [21,22]. The second advantage lies in the noninvasive nature of adipose tissue collection and the fact that adipose tissue can be obtained in abundance from patients undergoing abdominoplasty for cosmetic or other medical purposes. ASCs do not express multihistocompatibility complex II and as a result they do not elicit an immune response and an inflammatory reaction in the recipient [23]. Therefore, ASCs enable the production of safe allogeneic or xenogeneic off-the-shelf urethral graft with consistent quality; which can solve the problem of graft availability.

Besides the lack of a desirable autologous urethral graft, the obscure characteristics of ECM produced by either DF or ASCs are the current research gaps in the field, which we planned to address in this research study. We hypothesized that the extracellular matrix produced by adipose-derived stem cells under optimal conditions can support the growth, proliferation and phenotype maintenance of seeded UCs and SMCs. Therefore, we aimed to characterize and improve the existing model of ASC-based self-assembled scaffold in terms of thickness and to validate the capability of ASC-based self-assembled scaffold in supporting the growth and phenotype maintenance of terminally differentiated status by seeded UCs and SMs, the two critical cell types in human urethral tissue.

## 2. Results

### 2.1. Cell Morphology, Cell Yield and Population Doubling Time 

**Figure 1:**
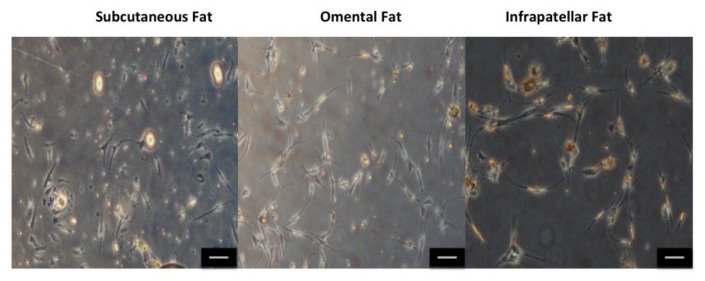
Phenotype of isolated adipose-derived stem cells (ASCs) from subcutaneous fat, omental fat and infrapatellar fat at P0. All cells show typical mesenchymal stem cell fibroblastic phenotype. No apparent difference in phenotype of isolated ASCs from three different sources of fat is detected. The scale bar represents 100 μm. Results are from a representative of three independent experiments.

**Figure 2:**
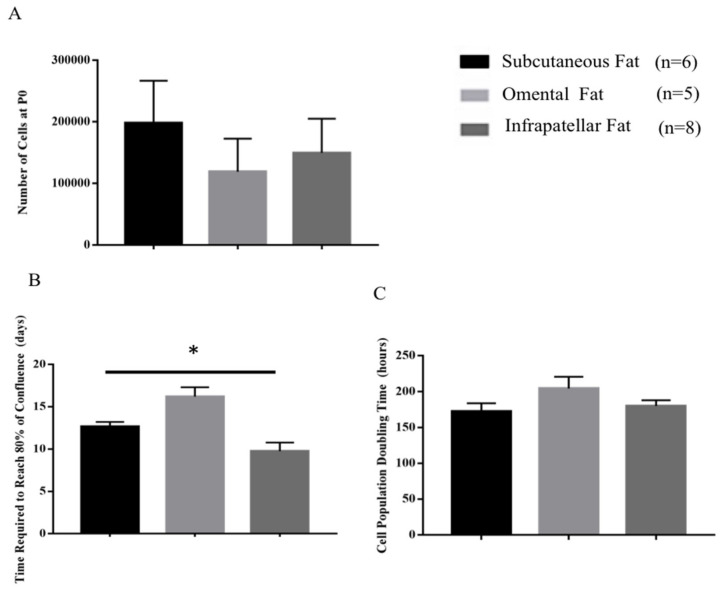
Cell yield at P0 (**A**), time required for ASCs reach to 80% confluency at P0 (**B**) and population doubling time (**C**). No significant differences were detected in cell yield and population doubling time among ASCs isolated from three of the fat sources. Isolated ASCs from infrapatellar fat required shorter time to reach 80% of confluency compared to two other sources. All graphs show mean measurements ± SEM. The results are representative of measurements from six (subcutaneous fat), five (omental fat) and eight (infrapatellar fat) biologically independent samples. * Represents statistically significant difference across three sources using one-way ANOVA (*p* < 0.05).

Figure 1 shows cell morphology with typical mesenchymal stem cell fibroblastic phenotype and no apparent differences in between phenotype of isolated ASCs from three different fat sources. Figure 2A shows the number of isolated ASCs (cell yield) from subcutaneous fat (197,833.33 ± 69,139.67), omental fat (119,000 ± 53,953.68) and infrapatellar fat (149,625 ± 55,497.08) at the end of P0; however, the differences between the cell numbers are not statistically significant. Figure 2B shows that ASCs isolated from infrapatellar fat, required significantly (*p* < 0.05) shorter time (days) (9.7 ± 1.03) to reach 80% confluency as compared to subcutaneous fat (12.66 ± 0.55) and omental fat (16.2 ± 1.11). However, population-doubling time (hours) (Figure 2C) of ASCs isolated from subcutaneous fat (172.6 ± 11.33), omental fat (204.48 ± 16.32) and infrapatellar fat (180.06 ± 8.05) showed no statistically significant difference among three different fat sources. For production of ASC-based scaffold, adipose tissue from subcutaneous fat had been selected as the favorable source because it was more readily available and abundant as compared to infrapatellar and omental fat.

### 2.2. Thickness Optimization Of ASC-Based Self-Assembled Scaffold under Different Parameters

#### 2.2.1. Different Seeding Densities and under Normoxic (21% O_2_) and Hypoxic (1% O_2_) Culture Conditions

**Figure 3:**
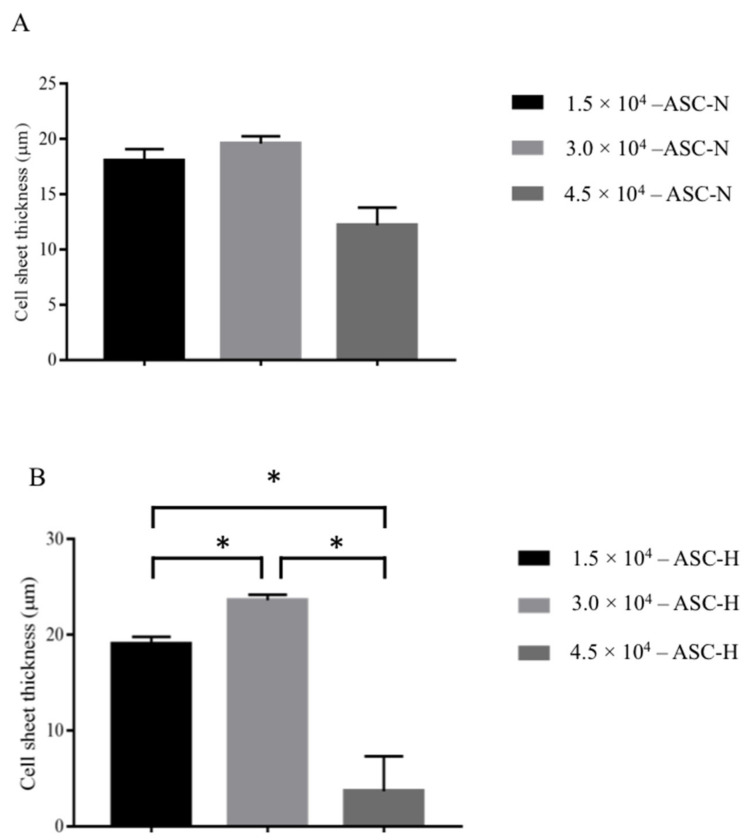
Thickness measurement of produced ASC-based self-assembled scaffold using 1.5, 3.0 and 4.5 × 10^4^ cells/cm^2^ cell seeding densities under normoxic (**A**) and hypoxic (**B**) culture conditions. The graph shows mean measurements ±SEM. The reading for thickness measurement for each of the self-assembled scaffolds was repeated in five predetermined positions (technical replicate). The results are representative of measurements from three biologically independent samples. * Represents statistically significant difference using paired t test (*p* < 0.05).

Figure 3A shows that under normoxic culture condition, the thickest measurement for ASC-based self-assembled scaffold (n = 3) had been achieved at 3.0 × 10^4^ cells/cm^2^ cell seeding densities with 19.6 ± 0.66 μm thickness as compared to 1.5 × 10^4^ cells/cm^2^ cell seeding densities with 18.06 ± 1.03 μm and 4.5 × 10^4^ cells/cm^2^ cell seeding densities with 12.2 ± 1.61 μm thickness measurements. However, the paired t test analysis showed that the differences between thicknesses are not statistically significant (*p* > 0.05). Figure 3B shows that under hypoxic culture condition, the thickest measurement for ASC-based self-assembled scaffold (n = 3) had been achieved at 3.0 × 10^4^ cells/cm^2^ cell seeding densities with 23.60 ± 0.59 μm thickness as compared to 1.5 × 10^4^ cells/cm^2^ cell seeding densities with 19.6 ± 0.72 μm and 4.5 × 10^4^ cells/cm^2^ cell seeding densities with 3.66 ± 3.66 μm thickness measurements. Paired t test analysis showed that the differences between thicknesses are statistically significant (*p* > 0.05). With 6 × 10^4^ cells/cm^2^ cell seeding density, in both hypoxic and normoxic culture conditions, ASCs-based self-assembled scaffold detached from the culture plate by day 7 (data is not shown). Therefore, 3 × 10^5^ cells/cm^2^ cell seeding density and hypoxic culture condition are the most favorable conditions for production of thickest ASC-based self-assembled scaffold.

#### 2.2.2. Different Concentrations of Ascorbic Acid

**Figure 4:**
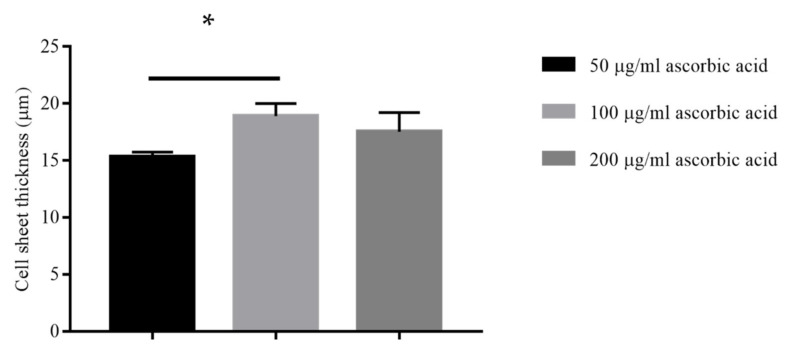
Thickness measurement of produced ASC-based self-assembled scaffold under hypoxic condition using 50, 100 and 200 μg/mL concentrations of ascorbic acid. The graph shows mean measurements ± SEM. The reading of thickness measurement for each of the self-assembled scaffolds was repeated in five predetermined positions (technical replicate). The results are representative of measurements from three biologically independent samples. * Represents statistically significant difference using paired t test (*p* < 0.05).

Figure 4 shows that the thickest measurement for ASC-based self-assembled scaffold (n = 3) had been achieved at 100 μg/mL of ascorbic acid concentrations with 20 ± 1.10 μm thickness as compared to 50 μg/mL of ascorbic acid concentrations with 15.33 ± 0.40 μm thickness and the paired t test analysis showed that the difference in thickness measurements was statistically significant (*p* < 0.05). Further increasing the concentration of ascorbic acid to 200 μg/mL resulted in decreasing the thickness of produced ASC-based self-assembled scaffold to 17.53 ±1.69 μg/mL with no statistically significant difference (*p* > 0.05) as compared to 100 μg/mL of ascorbic acid. Concentrations of 300 and 400 μg/mL of ascorbic acid were found to be toxic for the cultured ASCs since the cells started to detach from culture plate at day 14 of experiment initiation (data are not shown). Therefore, 100 μg/mL of ascorbic acid concentration is the most favorable condition for production of thickest ASC-based self-assembled scaffold.

#### 2.2.3. Different Mechanical Stimulation

**Figure 5:**
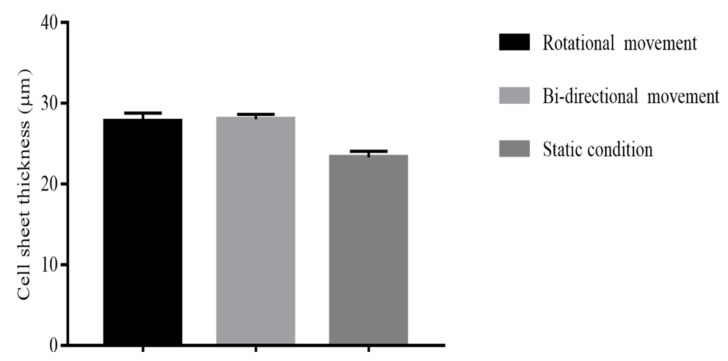
Thickness measurement of produced ASC-based self-assembled scaffold using rotational, bidirectional and static culture conditions. The graph shows mean measurements ± SEM. The reading of thickness measurement for each of the self-assembled scaffolds was repeated in five predetermined positions (technical replicate). The results are representative of measurements from three biologically independent samples. * Represents statistically significant difference using paired t test (*p* < 0.05).

Figure 5 shows that the thickness of ASC-based self-assembled scaffold (n = 3) had been increased in rotational culture condition with 27.8 ± 1.0 μm thickness and bidirectional culture condition with 28.06 ± 0.59 μm thickness as compared to static culture condition with 23.33 ± 0.76 μm thickness and the paired t test analysis showed that the difference in thickness measurements in rotational and bidirectional conditions were statistically significant (*p* < 0.05) as compared to static condition. However, the thickness of produced ASC-based self-assembled scaffold in rotational condition was found to have no statistically significant difference (*p* > 0.05) as compared to bidirectional condition. Therefore, mechanical culture condition (either rotational or bidirectional) is the most favorable condition for production of thickest ASC-based self-assembled scaffold.

### 2.3. Characterization Of ASC-Based Self-Assembled Scaffold

#### 2.3.1. Phase Contrast Imaging

**Figure 6:**
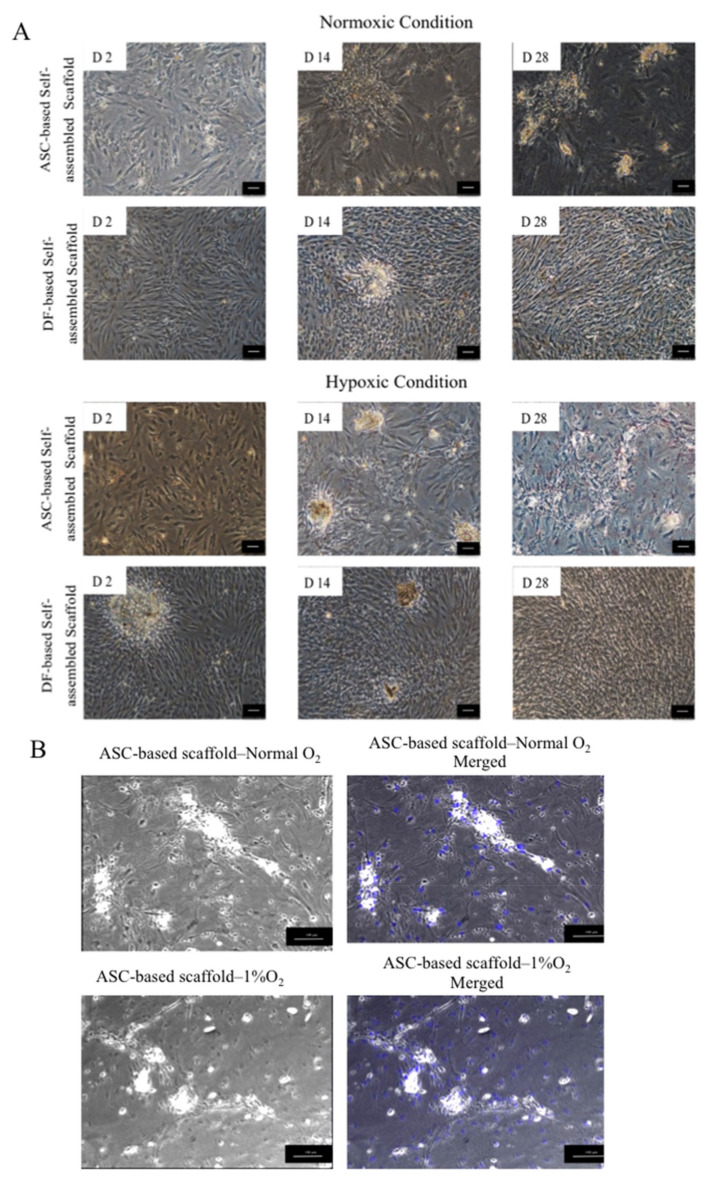
(**A**) Phase contrast imaging of ASC and dermal fibroblast (DF)-based self-assembled scaffolds. ASCs and DFs both remain attached and were proliferating during 28 days of culture period. D 2, D14 and D28, represents Day 2, Day 14 and Day 28, respectively. (**B**) In ASC-based self-assembled scaffold, 4′,6- diamidino-2-phenylindole (DAPI) was used as nuclear counterstain in blue color. Merged images show the absence of cell nuclei in the formed aggregates. The scale bar represents 100 μm. Results are from a representative of three independent experiments.

Figure 6A shows that in both normoxic and hypoxic conditions, ASCs similar to DFs proliferated and remained attached to the culture plate during 28 days of culture period. ASCs morphologically seemed to be flattened by the end of culture period while DFs could retain their thin and fibroblastic shape. In both normoxic and hypoxic conditions, ASCs and DFs responded to the introduced ascorbic acid by production of aggregates and secretion of compounds. Figure 6B clearly shows the absence of cell nuclei (blue color) in the formed aggregates, which indicates that the aggregates are not made from cell clumps, and most probably they are formed as a result of extracellular matrix components accumulation.

#### 2.3.2. Masson’s Trichrome and Immunocytochemical Staining

**Figure 7:**
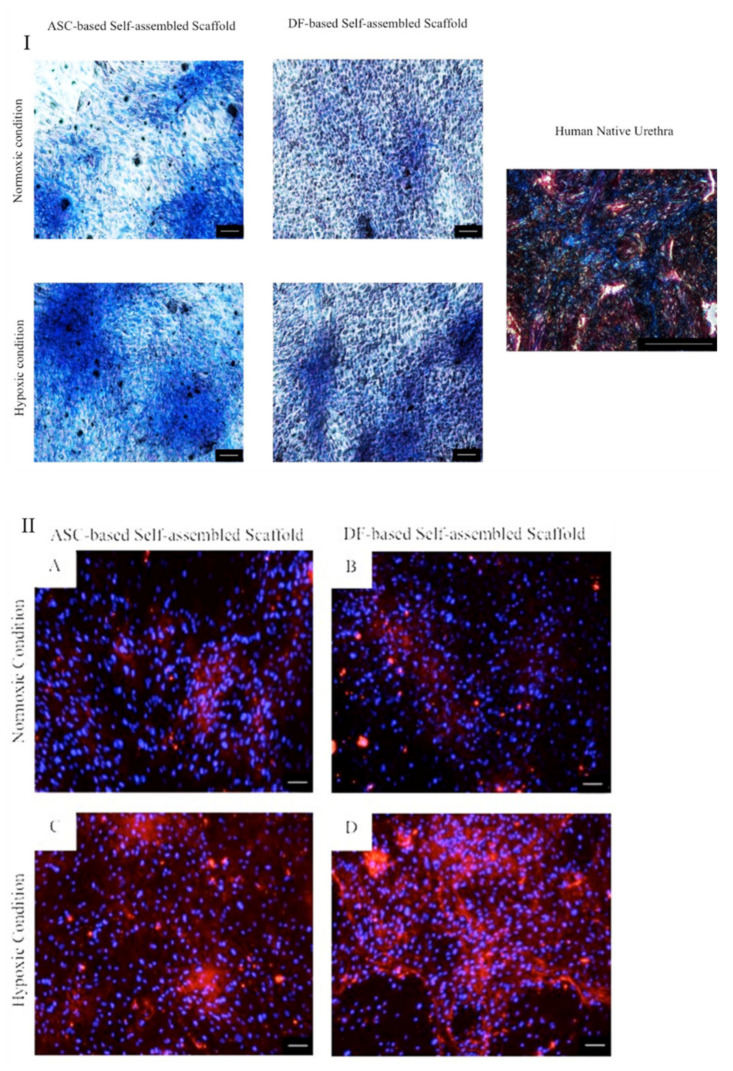
(**I**) Masson’s trichrome staining of ASC-based self-assembled scaffold in which the expression of collagen is detected in blue color. DF-based self-assembled scaffold was used as comparative control. Human native urethra was used as positive control. (**II**) Immunocytochemical staining of ASC-based self-assembled scaffold with anticollagen type I antibody in which the expression of collagen type I, is detected in red color (**A**,**C**). DF-based self-assembled scaffold was used as comparative control (**B**,**D**). 4’,6-diamidino-2-phenylindole (DAPI) was used as nuclear counterstain in blue color in all samples. The scale bar represents 100 μm. Results are from a representative of three independent experiments.

Results shows detection of collagen (Figure 7I) and collagen type I (Figure 7II) in ASC and DF-based self-assembled scaffolds. The expression pattern of collagen in normoxic condition for both ASC and DF-based self-assembled scaffolds was similar to that of their counterpart in hypoxic condition. However, the expression of collagen in hypoxic culture condition was more intense as compared to normoxic culture condition for both ASC and DF-based self-assembled scaffolds and this observation can be due to the contribution of hypoxic condition in stimulating collagen secretion by ASCs and DFs. Human native urethra was used as positive control (Figure 7I) in which detection of collagen and muscle fibers are presented in blue and red, respectively. Results are from a representative of three biologically independent experiments. 

#### 2.3.3. Gene Expression Data Analysis

List of detected ECM genes in ASC-based self-assembled scaffold with their fold changes and their regulation status (upregulated or downregulated) vs. DF-based self-assembled scaffold as comparative control is presented in Table 1. Among 84 genes associated with extracellular matrix and adhesion molecules, 82 and 75 genes were detected in ASC-based self-assembled scaffold and DF-based self-assembled scaffold, respectively. Table 2 lists the non expressed and weakly expressed genes which were remarked with Ct value ≥ 35 and 33 ≤ Ct < 35, respectively. In ASC-based self-assembled scaffold two genes: *Matrix metallopeptidase 13* (*collagenase 3*) and *Matrix metallopeptidase 7* (*matrilysin, uterine*) were not detected. Meanwhile the genes *Integrin, alpha M* (complement component 3 receptor 3 subunit), *Matrix metallopeptidase 15* (membrane-inserted), and *Selectin P* (granule membrane protein 140 kDa, antigen CD62) with 33 ≤ Ct < 35 were considered as weakly expressed. In DF-based self-assembled scaffold the following nine genes were not detected: *ADAM metallopeptidase* with thrombospondin type 1 motif 13, *ADAM metallopeptidase* with thrombospondin type 1 motif 8, *Catenin* (cadherin-associated protein) delta 2 (neural plakophilin-related arm-repeat protein), *Hyaluronan synthase 1*, *Matrix metallopeptidase 13* (*collagenase 3*), *platelet/endothelial cell adhesion molecule*, *Selectin E*, *Selectin L*, and *Selectin P* (granule membrane protein 140kDa, antigen CD62). The gene *Kallmann syndrome 1 sequence* with 33 ≤ Ct < 35 was also considered as weakly expressed. With an exception to *PECAM1* gene (*platelet/endothelial cell adhesion molecule*) (*p* < 0.05), all of the upregulated and downregulated genes associated with extracellular matrix and adhesion molecules in ASC-based self-assembled scaffold were comparable to the control (DF-based self-assembled scaffold) (*p* > 0.05). Some of the upregulated and downregulated genes exhibited large fold regulation numbers, however, a statistically significant difference could not be established. For ASC-based self-assembled scaffold, these genes are *Matrix metallopeptidase 7* (*matrilysin, uterine*) and *Integrin, alpha M* (complement component 3 receptor 3 subunit) and for DF-based self-assembled scaffold, these genes are *Catenin* delta 2 (cadherin-associated protein; neural plakophilin-related arm-repeat protein), *Hyaluronan synthase 1*, and *Selectin E*). Therefore, the change in their regulation is negligible. The findings suggest that no significant difference exists between extracellular matrixes of ASC-based self-assembled scaffold as compared to DF-based self-assembled scaffold in gene expression level except for *PECAM1*.

### 2.4. Biocompatibility of ASC-Based Self-Assembled Scaffold with Urothelial Cells and Smooth Muscle Cells

#### 2.4.1. Cell Morphology Evaluation

**Figure 8:**
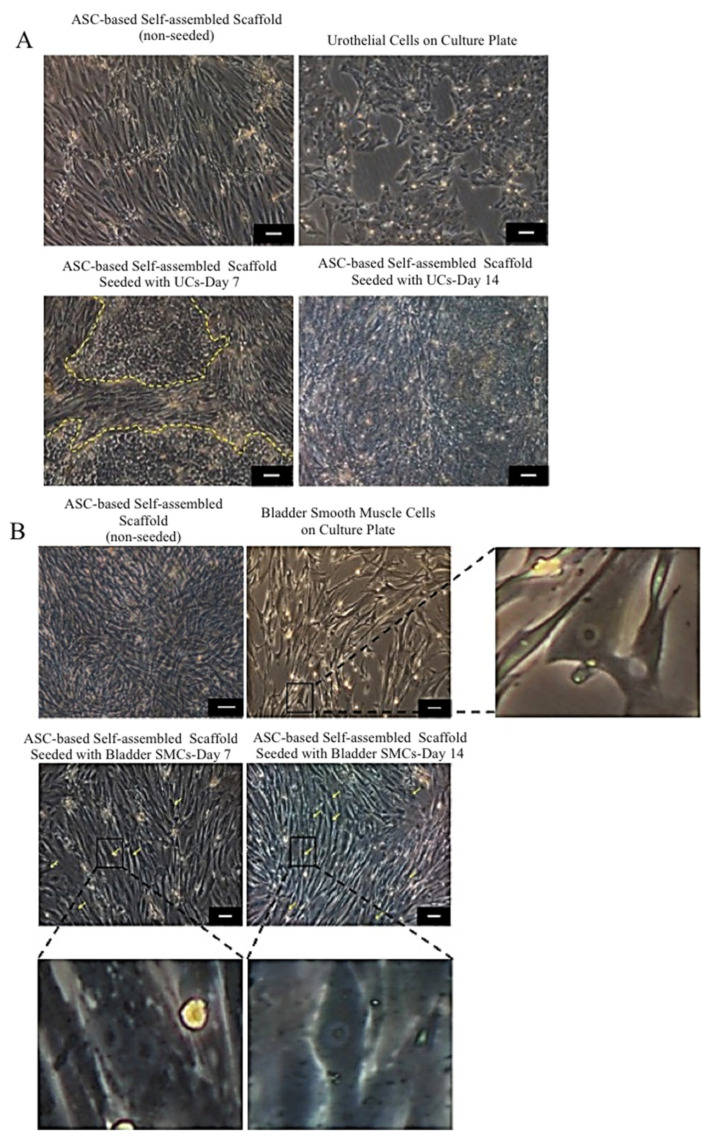
Growth evaluation of urothelial cells (UCs) (**A**), and smooth muscle cells (SMCs) (**B**) seeded on ASC-based self-assembled scaffold. Morphology and growth pattern of UCs and SMCs growing on the ASC-based self-assembled scaffold within 14 days of culture period are similar to that of UCs and SMCs seeded on the culture plate (control). Yellow dashed line marks UCs patches growing on ASC-based self-assembled scaffold (**A**). Distinctive large nucleus of SMCs growing on ASC-based self-assembled scaffold was detectable in some areas (yellow arrows). Enlarged photo of SMC with distinct nucleaus is also shown (**B**). The scale bar represents 100 μm. Results are from a representative of three independent experiments.

Seeded UCs (Figure 8A) and SMCs (Figure 8B) on the ASC-based self-assembled scaffold showed similar features and growth pattern to the UCs and SMCs seeded on the culture plate as control (native cells), respectively. Distinctive polygonal morphology of UCs compared to fibroblastic ASCs made it easy to visualize the growing UCs on top of ASC-based scaffold. The seeded UCs on the ASC based self-assembled scaffold were growing in a discrete patches pattern, were eventually expanded all over the surface area and could retain their native morphology (polygonal shape with regular dimensions) within 14 days of culture period. Both SMCs and ASCs shared similar fibroblastic features, however, the distinctive large nucleus of SMCs, could be clearly discerned in some areas (indicated with yellow arrows). This confirms the attachment and growth of SMCs on the ASC-based self-assembled scaffold. The seeded SMCs on the ASC-based self-assembled scaffold could retain their native fibroblastic morphology within 14 days of culture period. Results are from a representative of three independent experiments.

#### 2.4.2. Urothelial and Smooth Muscle Cells Markers Expression

**Figure 9:**
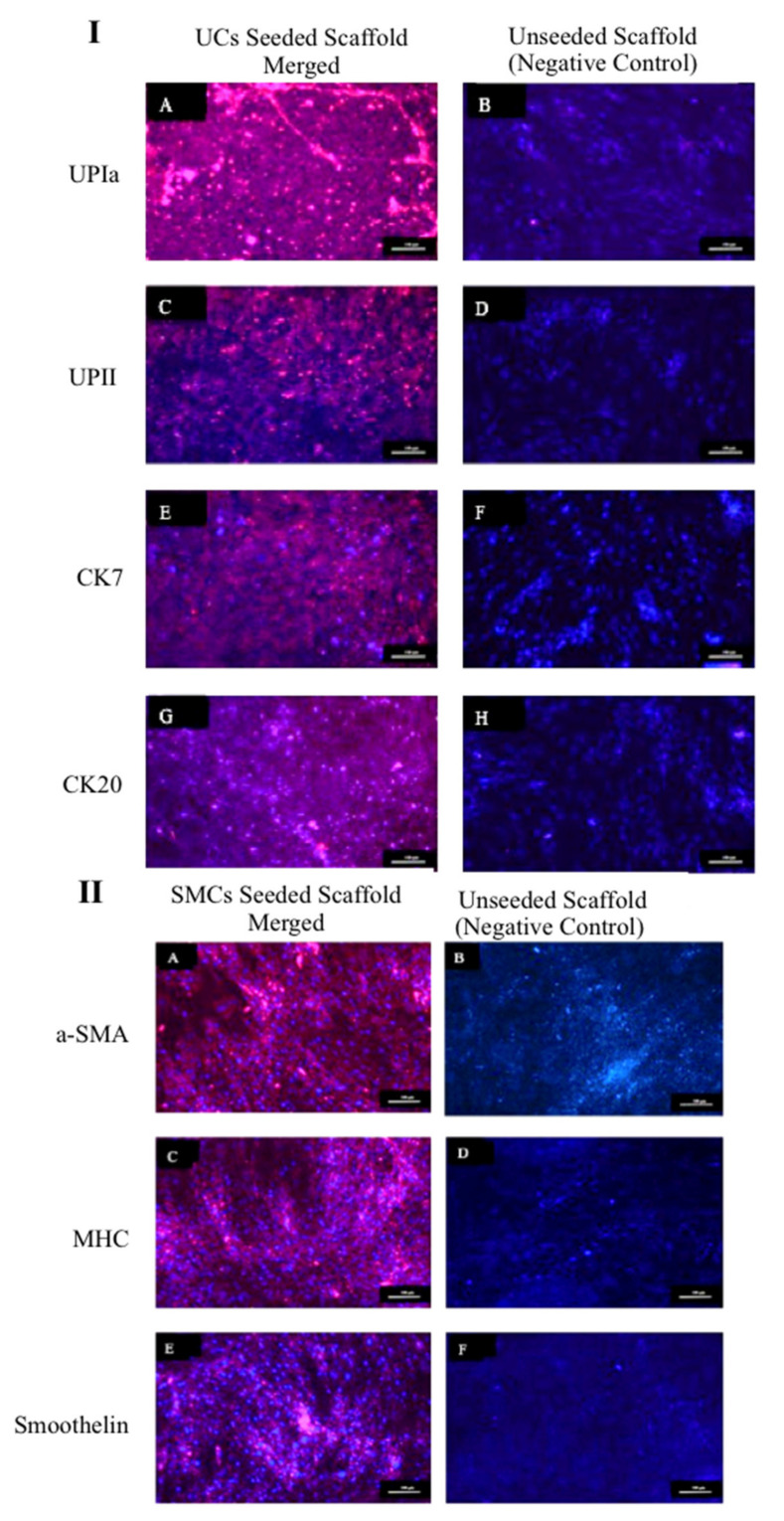
Immunocytochemical staining of seeded UCs (**I**) and SMCs (**II**) on ASC-based self-assembled scaffold. The expression of UPIa, UPII, CK7, and CK20 by UCs (IA,IC,IE,IG) and a-SMA, MHC and smoothelin by SMCs (IIA,IIC,IIE), are detected in red color. No UC and SMC marker expression was detected in negative control. 4’,6-diamidino-2-phenylindole (DAPI) was used as nuclear counterstain in blue color. The blue color detected in negative control (I and II), represents nuclei of UC (IB,ID,IF,IH) and ASCs (IIB,IID,IIF). The scale bar represents 100 μm. Results are from a representative of three independent experiments.

The expression of markers UPIa, UPII, CK7, and CK20 by UCs and expression of markers αSMA, MHC, and smoothelin by SMCs while seeded on the ASC-based self-assembled scaffold were detected (red color) and are shown in Figure 9I,II, respectively. ASC-based self-assembled scaffolds without being seeded with UCs or SMCs were used as negative control. In all negative controls, no expression of UC or SMC markers were detected, indicating the specificity of detected expressions to UCs and SMCs only. The findings suggest that ASC-based self-assembled scaffold can maintain UCs and SMCs specific markers for at least 14 days. Cell nuclei are counterstained with 4’,6-diamidino-2-phenylindole (DAPI) in blue color. Results are from a representative of three biologically independent experiments.

## 3. Discussion

### 3.1. Identifying the Ideal Source of Adipose-Derived Stem Cells

To commence this study, we investigated the best source of adipose tissue in terms of growth profile, from different anatomical locations: subcutaneous, omental and infrapatellar fat for isolating adipose-derived stem cells (ASCs). Significantly (*p* < 0.05) longer duration for ASCs from subcutaneous and omental fat to reach 80% confluence at passage 0 (Figure 2B) as compared to ASCs from infrapatellar fat, denotes a shorter growth rate at the initial stages of cell proliferation for ASCs from subcutaneous and omental fat as compared to ASCs from infrapatellar fat. This result might be due to tissue harvesting technique in which mechanical stress (liposuction) and heat (thermal knife) are introduced to the cells from subcutaneous and omental fat, respectively. Such extreme measures can adversely affect the viability and quality of the harvested tissue and subsequently reduce the growth rate of the isolated ASCs at the initial stages of culture. However, at passage 1, the differences in cell yield and cell population doubling time (Figure 2A,C) among the three adipose tissue types were not statistically significant (*p* > 0.05). Hence all three fat sources are feasible for isolating ASCs. However, in terms of tissue accessibility and abundance, subcutaneous fat is the preferred choice. 

### 3.2. ASC-Based Self-Assembled Scaffold Thickness Optimization

#### 3.2.1. Effect of Cell Seeding Density and Oxygen Concentration on Scaffold Thickness

In normoxic condition, the self-assembled scaffold reached to their maximum thickness at 1.5 × 10^4^ cells/cm^2^ seeding density, and further increasing cell seeding density to 3.0 × 10^4^ cells/cm^2^ did not have a significant effect of scaffold thickness (Figure 3A). A possible explanation is that ASCs reached their collagen secretion threshold (with 50 μg/mL ascorbic acid), or that the limited available growth surface restricts growth and proliferation of the cells. In hypoxic condition, the highest thickness of self-assembled scaffold was achieved at 3.0 × 10^4^ cells/cm^2^ cell seeding density (Figure 3B), even though the same limitations of growth surface availability persisted. Distler and colleagues (2007) showed that hypoxic condition directly contributed to the increased secretion and release of some major ECM proteins and that the longer exposure to hypoxic condition leads to higher upregulation of the genes encoding ECM proteins [24]. Therefore, our finding might have been due to the contribution of low O_2_ concentration [25], which promotes synthesis and secretion of collagen and ECM [26]. At higher cell seeding densities (4.5 and 6.0× 10^4^ cells/cm^2^), detachment of the cells from culture plate could be due to insufficient nutrients and/or O_2_ for cell growth, or the excessive secretion of ECM by the cells, which resulted in detachment of the cells/self-assembled scaffold from the culture plate.

#### 3.2.2. Effect of Ascorbic Acid Concentration on Scaffold Thickness

Ascorbic acid is one of the cofactors in synthesizing collagen from its precursors [25], and it also facilitates release of accumulated procollagen within cells [27]. Ascorbic acid had been widely used as one of the reagents in mesenchymal stem cell osteogenic differentiation media [28], however; the effect of ascorbic acid on promoting the extracellular matrix deposition is among the most recent discoveries [29]. Therefore, this study was optimizing the concentration of ascorbic acid as one of the contributing factors, which controls extracellular matrix deposition. Our findings (Figure 4), which show significant (*p* < 0.05) increase in the thickness of ASC-based self-assembled scaffold upon increasing ascorbic acid concentration from 50 to 100 μg/mL can be due to the increased efficacy of collagen synthesis using the exact same amount of collagen precursors, rather than due to increased cell proliferation. We also found that ascorbic acid concentration of ≥300μg/mL is toxic to the ASCs because it resulted to detachment of ASCs from culture plate as early as day 14 upon initiation of cell culture. It was previously reported in literature that toxicity of ascorbic acid is due to excessive accumulation of H_2_O_2_. H_2_O_2_ is generated by the interaction of ascorbic acid with iron ions in the culture medium [30], and the case of excessive H_2_O_2_ production, the enzyme catalase is not able to digest all existing H_2_O_2_ into water and it ultimately leads to apoptosis and cell death. It is worth mentioning here that the reactive oxygen species (ROS), which are produced as a result of cellular response to oxidative stress, has been associated with graft failure [31]. Often, long-term exposure to hypoxia can induce formation of ROS [32]. On the other hand, application of ascorbic acid as an antioxidant reagent [33], helps in balancing and modulating ROS formation [32]. Therefore, the possible production of ROS during ASCs self-assembled scaffold production could be studied in future and whether the application of ascorbic acid mediates the ROS formation produced needs further investigation.

#### 3.2.3. Effect of Mechanical Stimulation on Scaffold Thickness

Cells alter their behavior in response to mechanical stimuli. Vascular smooth muscle cells [34], and bovine aortic endothelial cells [35], when placed under dynamic condition synthesize significantly more collagen and proteoglycans, respectively, as compared to static condition. Rousseau et al. (2013) and Maceau-Fortier et al. (2013) showed that ASC-based self-assembled scaffold produced in dynamic condition was thicker as compared to the one produced in static condition [19,36]. They also reported that dynamic condition did not change the total DNA content and hence did not promote cell proliferation. Therefore, the increase in thickness was most probably due to improved cell-mediated matrix deposition [36]. Consistent with previous reports, our findings (Figure 5) also show that mechanical stimulation resulted in a significant increase (*p* < 0.05) in the thickness of ASC-based self-assembled scaffold as compared to static condition. To the best of our knowledge, this is the first time the comparison between the effects of two types of dynamic conditions (rotational vs. bidirectional) on ASC-based self-assembled thickness has been reported. We have found that, as expected, mechanical stimulation increased the thickness of the scaffold as compared to dynamic condition; however, the type of mechanical stimulation (rotational vs. bidirectional) did not produce any significant difference in the end results. The optimal conditions (3 × 10^4^ cells/cm^2^, hypoxic condition, 100 μg/mL ascorbic acid, and dynamic culture condition), resulted in production of ASC-based self-assembled scaffold with thickness of 28.06 ± 0.59 μm which is higher than that of previously reported thickness [15], ranging from 20.21 to 23.28 μm.

### 3.3. Characterization of ASC-Based Self-Assembled Scaffold

Here, we aimed to fabricate an ASC-based self-assembled scaffold for replacing urethral connective tissue (lamina propia) and ideally with similar composition to the native tissue. In the best of our knowledge, only a few studies in the literature have focused on the components of the human urethra and they had reported presence of collagen type I and III [25], glycosaminoglycans [37], elastic fibers and proteoglycans [38], in the ECM of human urethra. Previous studies had also reported detection of collagen type I and III [16], elastin [17], fibronectin and laminin [19], in DF-based self-assembled scaffold and detection of collagen type I, III, fibronectin, laminin [19], and elastin [15], in ASC-based self-assembled scaffold.

Consistent with the literature, we detected collagen and collagen type I in particular (Figure 7I,II, respectively), in both ASC and DF-based self-assembled scaffold. The expression profile of genes associated with ECM and adhesion molecules (Table 1) show detection of *collagen type I*, *fibronectin* and *laminin* in both scaffolds. With the exception of the *PECAM1* gene (*platelet/endothelial cell adhesion molecule*), which was significantly (*p* < 0.05) upregulated in ASC-based self-assembled scaffold as compared to DF-based self-assembled scaffold, all of the detected genes associated with ECM and adhesion molecules in the ASC-based self-assembled scaffold were comparable to that of the DF-based self-assembled scaffold. PECAM 1 has a molecular mass of 130 kDa and mediates interactions between leukocytes and endothelial cells and regulates vascular permeability. The adhesive property of PECAM 1 facilitates the interaction of hemophilic PECAM1/PECAM1 by increasing PECAM 1 concentrations at the endothelial cell–cell border, when it functions as a regulator of the vascular permeability barrier and leukocyte trafficking [39]. Among the genes which were weakly expressed (33 ≤ Ct < 35) in ASC-based self-assembled scaffold, *selectin P* mediates interaction of endothelial or platelets with leukocytes and its biological importance is on the pathogeneses of inflammation, thrombosis and the growth and metastasis of cancers [40]. *Integrin alpha M* involves in various adhesive interactions of monocytes, macrophages and granulocytes as well as mediating the uptake of pathogens [41], and *matrix metallopeptidase 15* involved in breakdown of extracellular matrix in normal physiological processes such as reproduction and tissue remodeling [42]. The gene *Kallmann syndrome 1 sequence* with 33 ≤ Ct <35 was also considered as weakly expressed in DF-based self-assembled scaffold. The *Kallmann syndrome 1 sequence* gene, which is also known as *KAL 1* gene, was found to be involved in motility and migration of different neural cell types during development before birth. It is also involved in migration of nerve cells and the outgrowth and branching of axons and regulating the contact and adhesion of nerve cells [43]. Whether those genes have other undiscovered functions is unknown; however, from what has already been reported in literature it seems that *PECAM 1* gene and none of the weakly expressed genes have function which might interfere with the ASC or DF-based self-assembled scaffold suitability for urologic application. Further analysis of protein content of the ECM produced by ASCs and DFs is needed in order to reach a concrete conclusion and this remains as limitation of our study. However, our preliminary findings here on gene expression level, suggest that there is no significant difference between the ECM of the ASC-based and DF-based self-assembled scaffolds and, in that sense, ASC-based self-assembled scaffold can be used as a suitable substitute to the DF-based self-assembled scaffold 

Even though the best approach would be to compare the ECM components of the ASC-based self-assembled scaffold with the native ECM of human urethra, such tissue was unavailable, so we compared it with the ECM produced by DF-based scaffold, as of commonly used self-assembled scaffold in urologic studies.

### 3.4. Biocompatibility of the ASC-Based Self-Assembled Scaffold with Urothelial and Smooth Muscle Cells

The significance of the urothelium in its fully differentiated state is highlighted in terms of the importance of metabolic homeostasis. Matured urethra prevents passage of toxins, ions, and water between urine and the underlying tissues and blood. Most of urinary tract diseases are rooted in alternation of blood–urine barrier function and disturbed metabolic homeostasis. The barrier function of the urothelium is developed upon differentiation of basal UCs into superficial UCs [44]. Among the superficial UC markers uroplakins (UP) such UPIa, UPIb, UPII, and UP IIIa are the most significant ones, and through their functional organization they contribute to the impermeability of the urothelium [45]. Tracking the changes in the expression pattern of some cytokeratins (CKs) can also indicate UC differentiation status. As superficial UCs become fully differentiated, CK7 is developed primarily, followed by CK20; with the last being exclusively expressed in umbrella cells as a marker of highly and terminally differentiated superficial cells [44].

In previous study it was found that DF-based self-assembled scaffold could support the expression of CK8/18 (marker of transitional epithelium) by seeded UCs and formation of stratified urothelium [16]. It was also reported that DF-based self-assembled scaffold could support expression of UPII and CK20 by seeded UCs only when the tissue-engineered construct is placed under dynamic condition [17]. DF-based self-assembled scaffold, only when subjected to cyclic pressure, develops feature of impermeability to urea [18]. However, ASC-based self-assembled scaffold was found to be not supportive to the phenotype maintenance of seeded UCs. ASC-based self-assembled scaffold not alone, but only when it was used in hybrid with DF (ASC-DF-based self-assembled scaffold), supported the expression of UPIb, UPII and UPIII and allowed formation of transitional and well-differentiated urothelium [19].

As opposed to previous finding [19], we proved here (Figure 9I) that ASC-based self-assembled scaffold which is produced under optimal conditions (3 × 10^4^ cells/cm^2^, hypoxic condition, 100 μg/mL ascorbic acid, and dynamic culture condition), is able to support the expression of terminally differentiated UC markers (UPIa, UPII, CK7, CK20), while the UCs are cultured and maintained under static condition. Positive expression of UC specific markers, while seeded on the ASC-based self-assembled scaffold indicates that UCs are in their highly differentiated status, which correlates with urethral impermeability. These results might be associated to (i) the contribution of the hypoxic condition or mechanical stimulation during ECM formation by ASCs, or (ii) the expression of laminin 5 in the ASC-based self-assembled scaffold which has been postulated to have a critical role in establishing the urothelium [19].

We also demonstrated here (Figure 9II) the capability of ASC-based self-assembled scaffold in supporting the expression of specific markers in SMCs; the α-SMA (marker of SMC phenotype at its initial stage), MHC (specific marker of SMC lineage in later stages of development [46]) and Smoothelin (marker of fully differentiated contractile SMCs [47]), while being seeded on the scaffold and this further indicates the suitability of the ASC-based self-assembled scaffold for urologic studies. Moreover, ASCs were found to maintain their undifferentiated status in vivo [48], therefore, ASC-based self-assembled scaffold continues to exert its favorable paracrine properties such as anti-inflammatory and angiogenic effects [49], upon implantation. This further emphasizes the superiority of ASC-based self-assembled scaffold for regenerative and reconstructive purposes in general.

## 4. Materials and Methods

### 4.1. Cell Isolation and Culture

Human tissues were collected after obtaining each patient’s written consent and all protocols were approved by the institutional research and ethics committee (UKM PPI/111/8/JEP-2017-186). Human skin biopsies were collected from patients undergoing abdominoplasty and human adipose tissues were collected from patients undergoing abdominoplasty (subcutaneous fat), sleeve gastrectomy (omental fat) and total knee replacement (infrapatellar fat). To isolate human dermal fibroblasts (DF) [50,51], skin sample was washed with Dulbecco’s phosphate buffer saline (1×) (DPBS, Invitrogen, Waltham, MA. USA), minced into small pieces and digested with 0.6% collagenase type I (Worthington, Lakewood, NJ, USA) for 4–5 h at 37°C in a shaker incubator. The cell suspension was then centrifuged at 5000 rotation per minute (rpm) followed by seeding and maintaining the cells in 1:1 Ham’s F12 nutrient: Dulbecco’s modified eagle medium (F12:DMEM) (Gibco, Waltham, MA, USA) supplemented with 10% (*v/v*) fetal bovine serum (FBS) (Biowest, Riverside, MO, USA) and 1% (*v*/*v*) antibiotic-antimycotic (AA) (Gibco, Waltham, MA, USA) (DMEM-F12 complete medium). To isolate human adipose-derived stem cells (ASC), adipose tissue was washed with 1× DPBS (Invitrogen, Waltham, MA, USA), minced into small pieces and digested with 0.6% collagenase type I (Worthington, Lakewood, NJ, USA) for 1 h at 37 °C in a shaker incubator. The cell suspension was then centrifuged at 5000 rpm followed by seeding and maintaining the cells in DMEM-F12 complete medium. Cells were maintained at 37 °C in a 5% CO_2_ incubator, with medium changes every 2–3 days. Once cells reached 70–80% confluence, cell dissociation was performed using 0.05% Trypsin-EDTA (Gibco, Waltham, MA, USA).

### 4.2. Cell Growth Profile Assessment 

To determine the best source of ASCs in terms of growth profile, the cell yield, time (days) required to reach 80% confluence at passage 0 (P0) and the cell population doubling time was studied. P0 cells are defined as fresh cells (ASCs), which are firstly attached to the cell culture plate upon isolation from the source tissue. Therefore, ASCs were isolated from 3 mL of adipose tissue from three different anatomical sources including subcutaneous fat (six biological sample), omental fat (five biological samples) and infrapatellar fat (eight biological samples). The isolated cells were cultured and maintained in a 6-well plate (Greiner Bio-one, Frickenhausen, Germany) as previously described (Section 4.1). The number of cells at the end of P0 and P1 were used to calculate cell yield and to plot the population doubling time. One-way ANOVA was used for statistical analysis. Cell doubling time was calculated using an online doubling time calculator following the formula below:

$$X=\;\frac{h\;\times \;\ln\;\left(2\right)}{\ln\;\left(\frac{c2}{c1}\right)}$$

where c is the number of cells at each time of collection and ln is a neperian logarithm (Roth, 2006, http://www.doubling-time.com/compute.php <accessed on February 2018>).

### 4.3. Production of ASC-Based Self-Assembled Scaffold under Different Conditions

Production of ASC-based self-assembled scaffold under different conditions was performed for the purpose of identifying the best parameters for production of thickest possible scaffold. The optimal condition(s) in each step was retained and used in the optimization of the next parameter.

#### 4.3.1. Different Seeding Densities and under Normoxic (21% O_2_) and Hypoxic (1% O_2_) Culture Conditions

ASCs at P3 were seeded in two sets of 6-well tissue culture plates with three different cell seeding densities including 1.5 × 10^4^, 3 × 10^4^ and 4.5 × 10^4^ cells/cm^2^. Cells were cultured in DMEM-F12 complete medium supplemented with 50 μg/mL of ascorbic acid (Sigma-Aldrich, St. Louis, MO, USA). One set of cells was incubated in 37 °C in 5% CO_2_ incubator, normoxic (21% O_2_) condition and the other set of cells was incubated in hypoxic (1% O_2_) condition. The media was replaced every other day for 28 days. For each cell seeding density, the experiment was repeated with three biologically independent samples.

#### 4.3.2. Different Concentrations of Ascorbic Acid

ASCs at P3 with 3 × 10^4^ cells/cm^2^ cell seeding density were seeded in a 6-well plate and were cultured in DMEM-F12 complete medium supplemented with different concentrations of ascorbic acid including 0, 50, 100, and 200 μg/mL. Cells were then incubated at 37 °C in a 5% CO_2_ incubator in hypoxic (1% O_2_) condition. The media was replaced every other day for 28 days. For each ascorbic acid concentration, the experiment was repeated with three biologically independent samples.

#### 4.3.3. Different Mechanical Stimulation 

**Figure 10:**
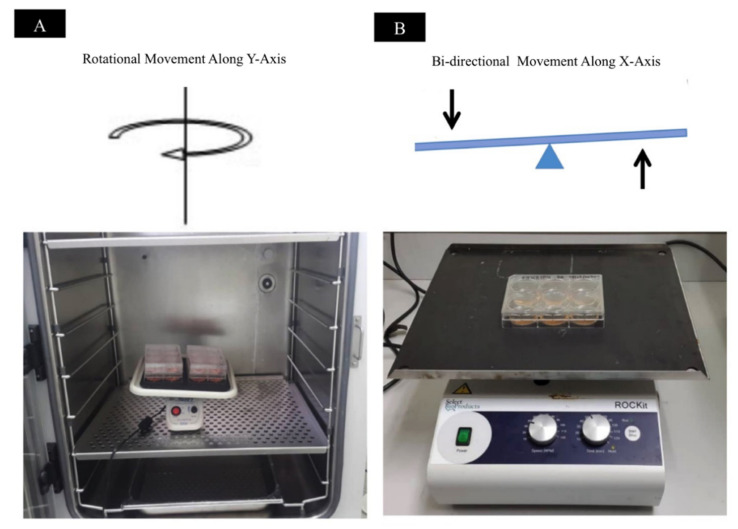
Mechanical stimulation introduced onto ASC-based self-assembled scaffolds in culture. (**A**) Shows rotational movement along the *Y*-axis and (**B**) shows bidirectional movement along the *X*-axis.

ASCs at P3 with 3 × 10^4^ cells/cm^2^ cells seeding density were seeded in three sets of 6-well tissue culture plate. Cells were cultured in DMEM-F12 complete medium supplemented with 100 μg/mL of ascorbic acid. The cells were incubated in 37 °C in a 5% CO_2_ incubator in hypoxic (1% O_2_) condition with media being changed every other day, for 28 days. Before introducing the mechanical stimulation, seeded cells were allowed to attach to the culture plate in static condition for 24 h. Then, one set of cells was subjected to rotational movement of 35 rpm along the Y-axis and the other set of cells was subjected to bidirectional movement with 35 rpm along X-axis. Additionally, one set of cells was cultured in static condition. For each of the mechanical conditions, the experiment was repeated with three biologically independent samples. Figure 10 shows graphical illustration of method applied for introduction of mechanical stimulation onto the cells.

### 4.4. Self-Assembled Scaffold Thickness Measurement 

#### 4.4.1. Immunocytochemical Staining 

To measure the thickness of self-assembled scaffold, expression of collagen type I in extracellular matrix produced by ASCs was detected. Hence, self-assembled scaffold was fixed in 4% (*w*/*v*) paraformaldehyde (PFA) (Sigma-Aldrich, St. Louis, MO, USA) for 20 min at 4 °C followed by three times washing with phosphate buffer saline (PBS)(1×) (Invitrogen, Waltham, MA, USA). Cell permeabilization was performed with 0.5% (*v*/*v*) triton-X (Sigma-Aldrich, St. Louis, MO, USA) with 20 min incubation at room temperature followed by three times washing with PBS (1×). Nonspecific binding was blocked using 10% (*v*/*v*) goat serum (Thermo Fisher, Waltham, MA, USA) at 37 °C for 1 h. Mouse monoclonal anti-collagen I antibody (COL-1/ab6308, Abcam, Cambridge, UK) with 1:200 dilution ration in 1% (*v*/*v*) goat serum was used as primary antibody at 4°C for overnight incubation in the dark. Cells then were washed three times with PBS (1×) followed by incubation with secondary antibody, goat anti-mouse Alexa Fluor 594 (Invitrogen, Waltham, MA, USA) with 1:300 dilution ration in 1% (*v*/*v*) goat serum at 37 °C for 2 h. The cells then were washed three times with PBS (1×) and were counterstained with 4’,6-Diamidino-2-Phenylindole, Dihydrochloride (DAPI) (Life Technologies, Carlsbad, CA, USA) diluted in PBS (1×) with 1:15,000 dilution ratio at room temperature for 20 min.

#### 4.4.2. Three-Dimensional Image Reconstruction and Thickness Measurement

**Figure 11:**
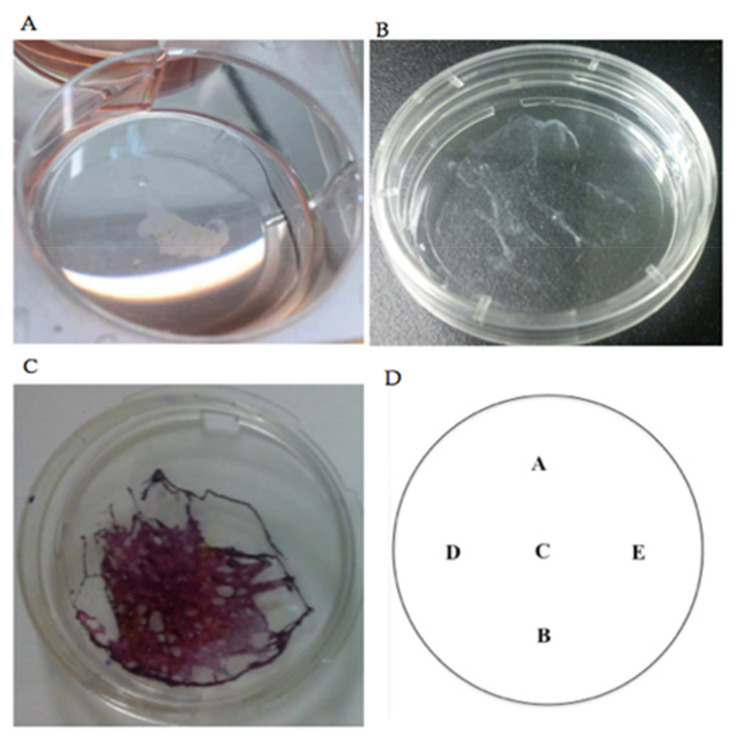
(**A**). Gross view of the floating ASC-based self-assembled scaffold in culture media after being scrapped from the culture plate. (**B**) ASC-based self-assembled scaffold upon transferring into a new culture plate. (**C**). To visualize the scaffold, it was stained with Masson’s trichrome stain. (**D**) Shows the five predetermined positions for measuring the thickness.

**Figure 12:**
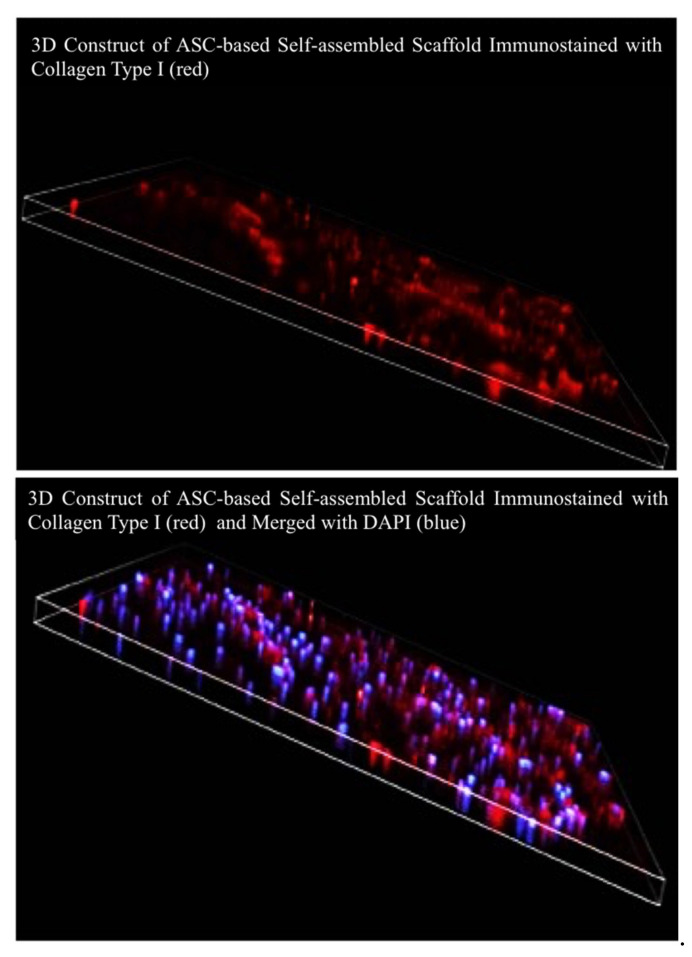
Graphical illustration of method used in measuring the thickness of self-assembled scaffold. The graph shows the 3D construct of ASC-based self-assembled scaffold, stained with collagen type I in red and merged with DAPI in blue (nucleus staining).

**Figure 13:**
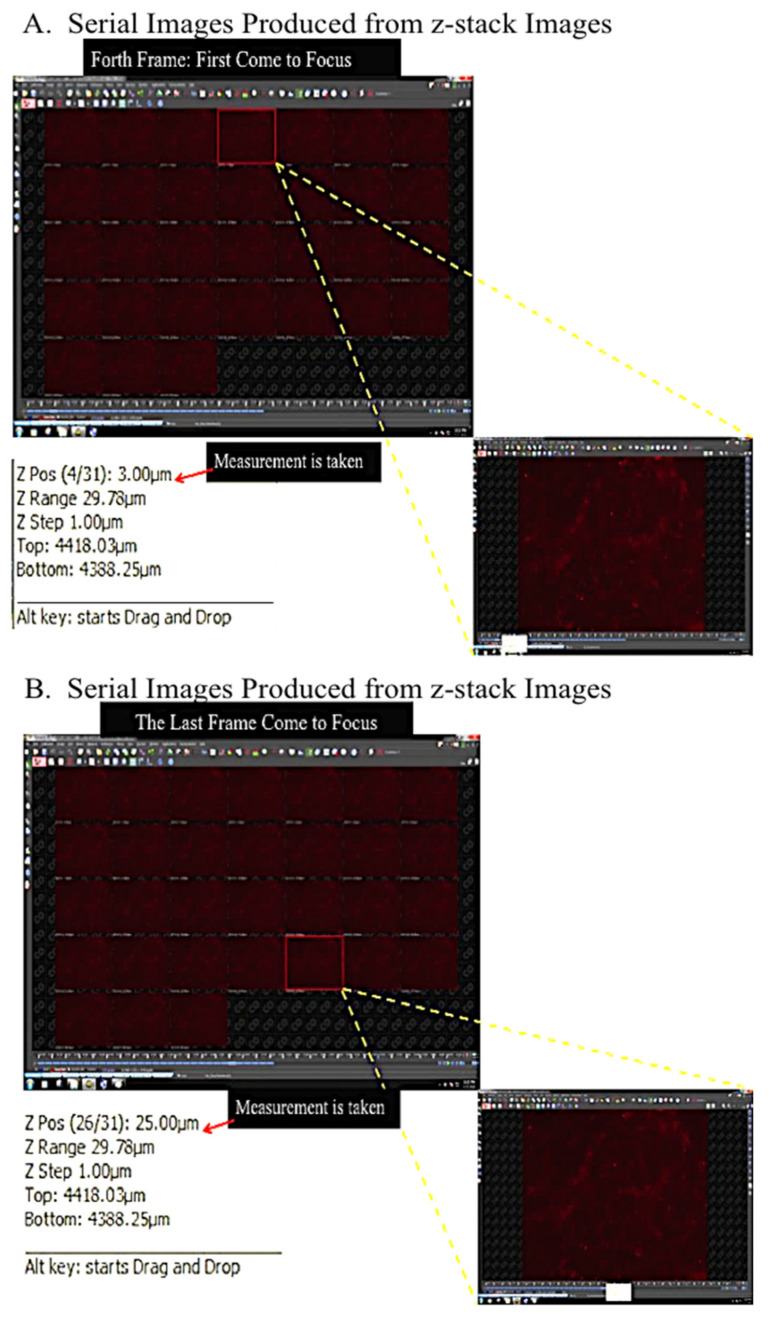
Serial images produced from Z-stack imaging. (**A**) The first and (**B**) the last frame that became in focus.

The stained self-assembled scaffold was visualized and evaluated using confocal microscope (Nikon A1, Tokyo, Japan) with Q Capture Pro 1.5 image analysis software (Olympus, Hamburg, Germany). To measure the thickness, Z-stack images with 1 μm optical slices were generated (three-dimensional (3D) image reconstruction). To be consistent in all measurements, 5 ms exposure time with 8.8 hardware gain were applied for all of the images. The reading of thickness measurement for each of the self-assembled scaffolds was repeated in five predetermined positions and the mean ±SEM was reported as the thickness of the respective self-assembled scaffold. For each of the experimental conditions (i.e., different cell seeding densities, different concentrations of ascorbic acid, etc.), the measurement was repeated with three biologically independent samples. To calculate the thickness of each self-assembled scaffold Equation (1) was followed. The paired t-test was used for statistical analysis and *p* < 0.05 was considered statistically significant. Figure 11A–C shows the gross view of ASC-based self-assembled scaffold and the arrangement of predetermined positions (Figure 11D) for thickness measurement. Figure 12 and Figure 13 illustrate the method applied in measuring the thickness.

(1) $$Thickness=thickness\;measurement\;of\;\left(last\;frame\;came\;into\;focouse-first\;frame\;came\;into\;focus\right)$$

### 4.5. Characterization of ASC-Based Self-Assembled Scaffold 

#### 4.5.1. Morphological Characterization

ASC-based self-assembled scaffolds were prepared in both normoxic (21% O_2_) and hypoxic (1% O_2_) culture conditions as previously described with 3 × 10^4^ cells/cm^2^ seeding density, 50 μg/mL of ascorbic acid (Sigma-Aldrich, St. Louis, MO, USA), supplementation and in static culture condition. At days 2, 14 and 28 a phase contrast image was made from each of the self-assembled scaffolds. To investigate the nature of aggregates appearing in self-assembled scaffolds, the cell nuclei were counterstained with DAPI (Life Technologies, Carlsbad, CA, USA). The stained self-assembled scaffolds were visualized and evaluated using confocal microscope (Nikon A1, Tokyo, Japan). DF-based self-assembled scaffolds were used as comparative control.

#### 4.5.2. Histological and Immunocytochemical Characterization 

ASC-based self-assembled scaffold was fixed in 4% (*w*/*v*) PFA (Sigma-Aldrich, St. Louis, MO, USA) for 20 min at 4 °C. Cells then were incubated in Bouin’s solution (Sigma-Aldrich, St. Louis, MO, USA) for 15 min at 56 °C, followed by washing with tap water. Nuclei staining was performed with incubation of the cells in Weigert’s iron hematoxylin solution (Sigma-Aldrich, St. Louis, MO, USA) for 5 min at room temperature, followed by washing with deionized water. Muscle fibers and cytoplasm staining was performed with incubation in Biebrich Scarlet-Acid Fuschin stain (Sigma-Aldrich, St. Louis, MO, USA) for 5 min at room temperature followed by washing with deionized water. Next, cells were incubated in phosphotungstic:phosphomolybic acid:deionized water solution (1:1:2) (Sigma-Aldrich, St. Louis, MO, USA) for 5 min at room temperature. Collagen staining was performed with incubation of the cells in Aniline Blue (Sigma-Aldrich, St. Louis, MO, USA) for 5 min at room temperature followed by incubation in 1% (*v*/*v*) acetic acid (Sigma-Aldrich, St. Louis, MO, USA) for 2 min at room temperature. Dehydration was performed with incubation of the cells in ascending concentrations of ethanol (Scharlau, Barcelona, Spain). Immunocytochemical staining was performed as previously described (Section 4.4.1). Human native urethra was used as positive control. DF-based self-assembled scaffolds were used as comparative control.

#### 4.5.3. Gene Expression Profile 

To detect the extracellular matrix components of ASC-based self-assembled scaffold in molecular level and to compare it with DF-based self-assembled scaffold, gene expression profiling was performed using the cataloged predesigned PCR (polymerase chain reaction) array of 84 genes (RT^2^ profiler PCR array for Human Extracellular Matrix and Adhesion Molecules, PAHS-013ZR, Qiagen, Hilden, Germany). Therefore, ASC and DF-based self-assembled scaffolds were produced with optimal culture conditions (3 × 10^4^ cells/cm^2^ cell seeding density, 1% O_2_, 100 μg/mL ascorbic acid and rotational movement with 35 rpm). For production of self-assembled scaffold, 28 days of culture period was needed; total RNA was extracted from the self-assembled scaffold at day 28 and stored at −80 °C. Complementary DNA synthesis and real-time quantitative polymerase chain reaction were carried out within 72 h from time of extraction to ensure the highest quality of RNA for analysis.

a. Total RNA extraction

For total RNA extraction, the RNeasy^®^ Mini Kit (Qiagen, Hilden, Germany) was used and the procedures were followed as per manufacturer’s instructions using 3–4 × 10^5^ cells as starting material. To eliminate genomic DNA contamination, on-column DNase digestion was performed using RNase-free DNase set (Qiagen, Hilden, Germany) and to elute extracted RNA, 40 μL of RNase-free water (Qiagen, Hilden, Germany) was added directly into the spin column membrane followed by centrifugation for 1 min at 14,000 rpm. The concentration and purity of extracted RNA was measured using a spectrophotometer (BioTek PowerWave XS, Winooski, VT, USA), while the integrity of the RNA was verified by 1% (*w*/*v*) agarose gel electrophoresis (Bio-rad, Hercules, CA, USA), where two distinct bands of 28S and 18S were detected.

b. Complementary DNA synthesis

Complementary DNA (cDNA) synthesis was carried out using RT^2^ First Strand Kit (Qiagen, Hilden, Germany) as per manufacturer’s instruction. Briefly, RNA template (800 ng) was mixed with genomic DNA removal reaction components and was incubated for 5 min at 42 °C. The resulting solution then was mixed with reverse-transcription mixture and the reaction was carried out in a Veriti Thermal Cycler (Applied Biosystems, Foster city, CA, USA) for exactly 15 min at 42 °C. Immediately, the incubation was carried out for further 5 min at 95 °C to facilitate the annealing, reverse-transcription and inactivation of the reaction.

c. Real-time quantitative polymerase chain reaction

As per manufacturer’s instruction, RT^2^ SYBR Green ROX FAST Mastermix (Qiagen, Hilden, Germany) was mixed with cDNA and nuclease-free water (Qiagen, Hilden, Germany). A total of 20 μL of prepared mixture was loaded into each well of the RT^2^ profiler PCR array for Human Extracellular Matrix and Adhesion Molecules, PAHS-013ZA (Qiagen, Hilden, Germany) using the QIAgility automated instrument (Qiagen, Hilden, Germany). The filled ring then was sealed using the Rotor-Disc Heat-Sealing Film (Qiagen, Hilden, Germany). Real-time quantitative polymerase chain reaction (RT-qPCR) was carried out on the Rotor Gene Q 100 (Qiagen, Hilden, Germany) with a holding time of 10 min at 95 °C, followed by 40 cycles of 10 s at 95 °C for denaturation step and 30 s at 60 °C for annealing/extension step. The threshold cycle (C_T_) value of 84 genes related to human extracellular matrix and adhesion molecules, five housekeeping genes, one genomic DNA control, three reverse transcription controls, and three positive PCR controls were determined. Fold changes (2^−ΔΔCT^) and *p* values between the comparative control group (DF-based self-assembled scaffold) and the experimental group (ASC-based self-assembled scaffold), from three biologically independent replicates were analyzed using Qiagen’s PCR array data analysis web portal (https://geneglobe.qiagen.com/us/analyze/ <accessed on June 2020>). To analyze gene expression data, threshold cycle (C_T_) cut-off value was set to 35 cycles and all C_T_ values were normalized against housekeeping genes (*Beta-2-microglobulin* (*B2M*), *Glyceraldehyde-3-phosphate dehydrogenase* (*GAPDH)* and *Hypoxanthine phosphor ribosyl transferase 1* (*HPRT1*)). Fold-change values greater than one indicates a positive or an up regulation and the fold-regulation is equal to the fold change. Fold-change values less than one indicates a negative or down regulation, and the fold-regulation is the negative inverse of the fold-change. The *p* values were calculated based on an unpaired Student’s t-test of the gene expression levels (2^−ΔCT^) for each gene in the experimental sample and the control and *p* < 0.05 was considered statistically significant.

### 4.6. Biocompatibility Assessment of ASC-Based Self-Assembled Scaffold with Human Urothelial Cells and Smooth Muscle Cells

#### 4.6.1. Growth Pattern Assessment

Human urothelial cell (UC) line (SV-HUC-1) was purchased from the American Type Culture Collection (ATCC, Manassas, VA, USA) and were revived, expanded and maintained in F12K complete medium (F12K medium (ATCC, Manassas, VA, USA), supplemented with 10% (*v*/*v*) FBS (Biowest, Riverside, MO, USA)), as per manufacturer’s recommendation. Human primary bladder smooth muscle cells (SMC, CC-2533) were purchased from Lonza (Lonza, Basel, Switzerland) and were revived, expanded and maintained in SmBM complete medium (SmBM™ Basal Medium (Lonza, Basel, Switzerland) supplemented with complimentary reagents (Lonza, Basel, Switzerland), including 1% (*v*/*v*) Insulin, 2% (*v*/*v*) hFGF-B (Human Hepatocyte Growth Factor-B), 1% (*v*/*v*) Gentamicin/Amphotericin-1000, 5% (*v*/*v*) FBS, and 1% (*v*/*v*) hEGF (Human Epithermal Growth Factor)), as per manufacturer’s recommendations. Two sets of ASC-based self-assembled scaffold were produced under optimal culture conditions (3 × 10^4^ cells/cm^2^ cell seeding density, 1% O_2_, 100 μg/mL ascorbic acid and rotational movement with 35 rpm). UCs at P34 with 2.5 × 10^3^ cells/cm^2^ seeding density were seeded on one set of produced ASC-based self-assembled scaffold. Low seeding density for UCs was used in order to avoid UCs detachment upon confluency and to allow them to sustain on top of the ASC-based self-assembled scaffold for at least 14 days. Cell seeded scaffold was cultured in 1:1 DMEM-F12 complete medium (Gibco, Waltham, MA, USA): F-12K complete medium (ATCC, Manassas, VA, USA), in 21% O_2_ (normxic) and the media was replaced every other day. Bladder SMCs at P4 with 7 × 10^3^ cells/cm^2^ seeding density were seeded on the second set of produced ASC-based self-assembled scaffold. Cell seeded scaffold was cultured in 1:1 DMEM-F12 complete medium (Gibco, Waltham, MA, USA):SmBM complete medium (Lonza, Basel, Switzerland), in 21% O_2_ (normoxic) and the media was replaced every other day. Pattern of UCs and SMCs growth on ASC-based self-assemble scaffold was evaluated using light microscope (Olympus, Hamburg, Germany) at days 7 and 14 upon cell seeding. The experiments were repeated with three biologically independent ASC-based self-assembled scaffolds.

#### 4.6.2. Urothelial Cells and Smooth Muscle Cells Specific Markers Expression

In order to evaluate the ability of ASC-based self-assembled scaffold in maintaining the phenotype of UCs and SMCs, production and seeding of ASC-based self-assembled scaffold with UCs and SMCs was performed as preciously described (Section 4.6.1). Immunocytochemical staining of seeded UCs and SMCs at day 14 was performed as previously described (Section 4.4.1). For UCs, the primary antibodies rabbit monoclonal anti-uroplakin Ia (UPIa-ab185970, Abcam, Cambridge UK) with 1:100 dilution ratio, rabbit monoclonal anti-uroplakin II (UPII-ab213655, Abcam, Cambridge, UK) with 1:300 dilution ratio, rabbit monoclonal anti-cytokeratin 7 (CK7-ab181598, Abcam, Cambridge, UK) with 1:100 dilution ratio, and rabbit monoclonal anti-cytokeratin 20 (CK20-ab76126, Abcam, Cambridge, UK) with 1:200 dilution were used. Goat anti-rabbit Alexa Fluor 594 (Invitrogen, Waltham, MA, USA) with 1:300 dilution ratios was used for secondary antibody staining. For SMCs, the primary antibodies mouse monoclonal anti-alpha-smooth muscle actin (αSMA-ab7817, Abcam, Cambridge, UK) with 5μg/mL dilution ratio, mouse monoclonal anti-smooth muscle myosin heavy chain (MHC-ab683, Abcam, Cambridge, UK) with 1:250 dilution ratio, and mouse monoclonal anti-smoothelin (ab21108, Abcam, Cambridge, UK) with 1:300 dilution ratio were used. Goat anti-mouse Alexa Fluor 488 (Invitrogen, Waltham, MA, USA), with 1:300 dilution ratio was used for secondary antibody staining. DAPI (Life Technologies, Carlsbad, CA, USA) stain was used for nuclei counterstaining. UCS and SMCs grown in a culture plate were used as positive control. ASC-based self-assembled scaffold without being seeded with UCs or SMCs was used as negative control. The stained samples were visualized using confocal microscope (Nikon A1, Tokyo, Japan). The experiment was repeated for three biologically independent ASC-based self-assembled scaffolds.

## 5. Conclusions

We present here for the first time the fabrication of an ASC-based self-assembled scaffold free from exogenous cells and that is entirely able to support the growth and phenotype of urothelial and smooth muscle cells. Our findings provide a foundation for future steps in the production of safe and efficient urethral graft to be used in urethral reconstructive surgeries, as an alternative to the current gold standards (buccal mucosa and genital skin). The best approach to tissue-engineering urethra is to mimic the native tissue. In our study, we used an ASC-based self-assembled scaffold as a urethral connective tissue replacement, and compared its components with that of the commonly used DF-based self-assembled scaffold. Ideally, we would compare the components of the fabricated scaffolds with that of connective tissue present in human native urethra. However, due to the unavailability of such data in the literature and the unavailability of human urethra from Hospital Universiti Kebangsaan Malaysia to perform the analysis, this remains as limitation of our study. The role and the implication of *PECAM 1* gene and whether its upregulation interferes with scaffold functionality and the proteomic profiling of ECM content produced by ASCs and DFs needs further investigation. Stacking of ASC-based scaffolds to construct a thicker sheet followed by seeding of UCs and SMCs on respective sides of the scaffold can be investigated. To promote integration of stacking ASC-based self-assembled scaffolds, culture parameters, such as application of serums from different origins, can be studied. Possibility and quality of multilayered urothelium formation of ASC-based scaffold and the permeability of such grafts also need further investigation. Moreover, since the initial ischemia upon grafting is believed to be the reason for graft contraction and fibrosis, it is suggested that a combination of endothelial cells and hypoxia-activated MSCs (known to promote angiogenesis and prevent inflammation) to be incorporated into the tissue-engineered construct.

## Figures and Tables

**Table 1:** Gene expression profile of ASC-based self-assembled scaffold and gene regulation status vs. DF-based self-assembled scaffold.

Position	Symbol	Gene Name	Fold Change	Fold Regulation
Genes Upregulated in ASC-Based Self-Assembled Scaffold vs. DF-Based Self-Assembled Scaffold				
A01	*ADAMTS1*	*ADAM metallopeptidase with thrombospondin type 1 motif, 1*	9.84	9.84
A02	*ADAMTS13*	*ADAM metallopeptidase with thrombospondin type 1 motif, 13*	66.87	66.87
A03	*ADAMTS8*	*ADAM metallopeptidase with thrombospondin type 1 motif, 8*	21.55	21.55
A04	*CD44*	*CD44 molecule (Indian blood group)*	13.88	13.88
A05	*CDH1*	*Cadherin 1, type 1, E-cadherin (epithelial)*	2.82	2.82
A06	*CLEC3B*	*C-type lectin domain family 3, member B*	42.51	42.51
A10	*COL14A1*	*Collagen, type XIV, alpha 1*	34.77	34.77
A12	*COL16A1*	*Collagen, type XVI, alpha 1*	1.67	1.67
B02	*COL4A2*	*Collagen, type IV, alpha 2*	5.51	5.51
B03	*COL5A1*	*Collagen, type V, alpha 1*	2.19	2.19
B06	*COL7A1*	*Collagen, type VII, alpha 1*	8.05	8.05
B09	*CTNNA1*	*Catenin (cadherin-associated protein), alpha 1, 102kDa*	8.05	8.05
B10	*CTNNB1*	*Catenin (cadherin-associated protein), beta 1, 88kDa*	2.36	2.36
B11	*CTNND1*	*Catenin (cadherin-associated protein), delta 1*	2.18	2.18
B12	*CTNND2*	*Catenin (cadherin-associated protein), delta 2 (neural plakophilin-related arm-repeat protein)*	1323.36	1323.36
C03	*HAS1*	*Hyaluronan synthase 1*	494.55	494.55
C05	*ITGA1*	*Integrin, alpha 1*	1.52	1. 52
C06	*ITGA2*	*Integrin, alpha 2 (CD49B, alpha 2 subunit of VLA-2 receptor)*	14.38	14.38
C07	*ITGA3*	*Integrin, alpha 3 (antigen CD49C, alpha 3 subunit of VLA-3 receptor)*	1.26	1.26
C11	*ITGA7*	*Integrin, alpha 7*	37.44	37.44
D05	*ITGB2*	*Integrin, beta 2 (complement component 3 receptor 3 and 4 subunit)*	31.26	31.26
D07	*ITGB4*	*Integrin, beta 4*	8.67	8.67
D09	*ANOS1*	*Kallmann syndrome 1 sequence*	72.84	72.84
D10	*LAMA1*	*Laminin, alpha 1*	1.27	1.27
D11	*LAMA2*	*Laminin, alpha 2*	1.93	1.93
D12	*LAMA3*	*Laminin, alpha 3*	2.89	2.89
E02	*LAMB3*	*Laminin, beta 3*	4.42	4.42
E09	*MMP14*	*Matrix metallopeptidase 14 (membrane-inserted)*	1.45	1.45
E10	*MMP15*	*Matrix metallopeptidase 15 (membrane-inserted)*	1.11	1.11
F04	*MMP9*	*Matrix metallopeptidase 9 (gelatinase B, 92kDa gelatinase, 92kDa type IV collagenase)*	5.40	5.40
F06	*PECAM1*	*Platelet/endothelial cell adhesion molecule*	160.52	160.52
F07	*SELE*	*Selectin E*	144.50	144.50
F08	*SELL*	*Selectin L*	17.2	17.2
F09	*SELP*	*Selectin P (granule membrane protein 140kDa, antigen CD62)*	7.2	7.2
F12	*SPG7*	*Spastic paraplegia 7 (pure and complicated autosomal recessive)*	238.85	238.85
G01	*SPP1*	*Secreted phosphoprotein 1*	3.69	3.69
G03	*THBS1*	*Thrombospondin 1*	1.22	1.22
G04	*THBS2*	*Thrombospondin 2*	5.85	5.85
G05	*THBS3*	*Thrombospondin 3*	82.90	82.90
G07	*TIMP2*	*TIMP metallopeptidase inhibitor 2*	2.66	2.66
G10	*VCAM1*	*Vascular cell adhesion molecule 1*	6.42	6.42
G11	*VCAN*	*Versican*	1.95	1.95
G12	*VTN*	*Vitronectin*	29.65	29.65
Total		43		
Genes Downregulated in ASC-Based Self-Assembled Scaffold vs. DF-Based Self-Assembled Scaffold				
A07	*CNTN1*	*Contactin 1*	0.93	−1.06
A08	*COL11A1*	*Collagen, type XI, alpha 1*	0.37	−2.70
A09	*COL12A1*	*Collagen, type XII, alpha 1*	0.09	−10.35
A11	*COL15A1*	*Collagen, type XV, alpha 1*	0.27	−3.61
B01	*COL1A1*	*Collagen, type I, alpha 1*	0.20	−4.81
B04	*COL6A1*	*Collagen, type VI, alpha 1*	0.52	−1.90
B05	*COL6A2*	*Collagen, type VI, alpha 2*	0.29	−3.40
B07	*COL8A1*	*Collagen, type VIII, alpha 1*	0.41	−2.38
B08	*CTGF*	*Connective tissue growth factor*	0.01	−70.93
C01	*ECM1*	*Extracellular matrix protein 1*	0.12	−8.11
C02	*FN1*	*Fibronectin 1*	0.05	−17.56
C04	*ICAM1*	*Intercellular adhesion molecule 1*	0.60	−1.66
C08	*ITGA4*	*Integrin, alpha 4 (antigen CD49D, alpha 4 subunit of VLA-4 receptor)*	0.44	−2.26
C09	*ITGA5*	*Integrin, alpha 5 (fibronectin receptor, alpha polypeptide)*	0.10	−9.71
C10	*ITGA6*	*Integrin, alpha 6*	0.57	−1.72
C12	*ITGA8*	*Integrin, alpha 8*	0.45	−2.19
D01	*ITGAL*	*Integrin, alpha L (antigen CD11A (p180), lymphocyte function-associated antigen 1; alpha polypeptide)*	0.37	−2.64
D02	*ITGAM*	*Integrin, alpha M (complement component 3 receptor 3 subunit)*	0.002	−384.89
D03	*ITGAV*	*Integrin, alpha V (vitronectin receptor, alpha polypeptide, antigen CD51)*	0.74	−1.33
D04	*ITGB1*	*Integrin, beta 1 (fibronectin receptor, beta polypeptide, antigen CD29 includes MDF2, MSK12)*	0.42	−2.36
D06	*ITGB3*	*Integrin, beta 3 (platelet glycoprotein IIIa, antigen CD61)*	0.08	−12.46
D08	*ITGB5*	*Integrin, beta 5*	0.22	−4.51
E01	*LAMB1*	*Laminin, beta 1*	0.04	−20.11
E03	*LAMC1*	*Laminin, gamma 1 (formerly LAMB2)*	0.35	−2.80
E04	*MMP1*	*Matrix metallopeptidase 1 (interstitial collagenase)*	0.36	−2.75
E05	*MMP10*	*Matrix metallopeptidase 10 (stromelysin 2)*	0.36	−2.71
E06	*MMP11*	*Matrix metallopeptidase 11 (stromelysin 3)*	0.03	−27.09
E07	*MMP12*	*Matrix metallopeptidase 12 (macrophage elastase)*	0.06	−15.41
E08	*MMP13*	*Matrix metallopeptidase 13 (collagenase 3)*	0.73	−1.35
E11	*MMP16*	*Matrix metallopeptidase 16 (membrane-inserted)*	0.12	−8.03
E12	*MMP2*	*Matrix metallopeptidase 2 (gelatinase A, 72kDa gelatinase, 72kDa type IV collagenase)*	0.45	−2.20
F01	*MMP3*	*Matrix metallopeptidase 3 (stromelysin 1, progelatinase)*	0.16	−5.99
F02	*MMP7*	*Matrix metallopeptidase 7 (matrilysin, uterine)*	0.003	−273.10
F03	*MMP8*	*Matrix metallopeptidase 8 (neutrophil collagenase)*	0.12	−8.03
F05	*NCAM1*	*Neural cell adhesion molecule 1*	0.42	−2.33
F10	*SGCE*	*Sarcoglycan, epsilon*	0.31	−3.16
F11	*SPARC*	*Secreted protein, acidic, cysteine-rich (osteonectin)*	0.67	−1.49
G02	*TGFBI*	*Transforming growth factor, beta-induced, 68kDa*	0.05	−18.80
G06	*TIMP1*	*TIMP metallopeptidase inhibitor 1*	0.23	−4.31
G08	*TIMP3*	*TIMP metallopeptidase inhibitor 3*	0.34	−2.86
G09	*TNC*	*Tenascin C*	0.67	−1.47
Total		41		

**Table 2:** List of the genes not expressed or weakly expressed in ASC-based and DF-based self-assembled scaffolds.

	Position	Symbol	Gene Name	*p* Value
**ASC-Based Self-Assembled Scaffold**				
Not Expressed (Ct ≥ 35)	E08	*MMP13*	*Matrix metallopeptidase 13 (collagenase 3)*	0.1179
	F02	*MMP7*	*Matrix metallopeptidase 7 (matrilysin, uterine)*	0.4480
Total	2			
Weakly Expressed (33 ≤ Ct < 35)	D02	*ITGAM*	*Integrin, alpha M (complement component 3 receptor 3 subunit)*	0.1161
	E10	*MMP15*	*Matrix metallopeptidase 15 (membrane-inserted)*	0.3720
	F09	*SELP*	*Selectin P (granule membrane protein 140kDa, antigen CD62)*	0.3630
Total	3			
**DF-Based Self-Assembled Scaffold**				
Not Expressed (Ct≥35)	A02	*ADAMTS13*	*ADAM metallopeptidase with thrombospondin type 1 motif, 13*	0.3750
	A03	*ADAMTS8*	*ADAM metallopeptidase with thrombospondin type 1 motif, 8*	0.4810
	B12	*CTNND2*	*Catenin (cadherin-associated protein), delta 2 (neural plakophilin-related arm-repeat protein)*	0.1145
	C03	*HAS1*	*Hyaluronan synthase 1*	0.3334
	E08	*MMP13*	*Matrix metallopeptidase 13 (collagenase 3)*	0.1179
	F06	*PECAM1*	*Platelet/endothelial cell adhesion molecule*	0.0003
	F07	*SELE*	*Selectin E*	0.2760
	F08	*SELL*	*Selectin L*	
	F09	*SELP*	*Selectin P (granule membrane protein 140kDa, antigen CD62)*	0.4390
Total	9			
Weakly Expressed (33 ≤ Ct < 35)	D09	*ANOS1*	*Kallmann syndrome 1 sequence*	0.3661
Total	1			
